# Molecular evolution of mammalian genes with epistatic interactions in fertilization

**DOI:** 10.1186/s12862-019-1480-6

**Published:** 2019-07-25

**Authors:** Claire C. Morgan, Michael W. Hart

**Affiliations:** 1 Department of Medicine, Imperial College London, London, W12 0NN UK; 2 Centre for Genomic Regulation (CRG), The Barcelona Institute of Science and Technology, Barcelona, Spain; 3 Department of Biological Sciences, Simon Fraser University, Burnaby, British Columbia V5A 1S6 Canada

**Keywords:** Gamete recognition, Zona pellucida, Positive selection, Coevolution, Sexual selection, Innate immunity

## Abstract

**Background:**

Genes that encode proteins associated with sperm competition, fertilization, and sexual conflicts of interest are often among the most rapidly evolving parts of animal genomes. One family of sperm-expressed genes (*Zp3r*, *C4bpa*) in the mammalian gene cluster called the regulator of complement activation (RCA) encodes proteins that bind eggs and mediate reproductive success, and are therefore expected to show high relative rates of nonsynonymous nucleotide substitution in response to sexual selection in comparison to other genes not involved in gamete binding at fertilization. We tested that working hypothesis by using phylogenetic models of codon evolution to identify episodes of diversifying positive selection. We used a comparative approach to quantify the evidence for episodic diversifying selection acting on RCA genes with known functions in fertilization (and sensitivity to sexual selection), and contrast them with other RCA genes in the same gene family that function in innate immunity (and are not sensitive to sexual selection).

**Results:**

We expected but did not find evidence for more episodes of positive selection on *Zp3r* in Glires (the rodents and lagomorphs) or on *C4BPA* in Primates, in comparison to other paralogous RCA genes in the same taxon, or in comparison to the same orthologous RCA gene in the other taxon. That result was not unique to RCA genes: we also found little evidence for more episodes of diversifying selection on genes that encode selective sperm-binding molecules in the egg coat or zona pellucida (*Zp2*, *Zp3*) in comparison to members of the same gene family that encode structural elements of the egg coat (*Zp1*, *Zp4*). Similarly, we found little evidence for episodic diversifying selection acting on two other recently discovered genes (*Juno*, *Izumo1*) that encode essential molecules for sperm–egg fusion.

**Conclusions:**

These negative results help to illustrate the importance of a comparative context for this type of codon model analysis. The results may also point to other phylogenetic contexts in which the effects of selection acting on these fertilization proteins might be more readily discovered and documented in mammals and other taxa.

**Electronic supplementary material:**

The online version of this article (10.1186/s12862-019-1480-6) contains supplementary material, which is available to authorized users.

## Background

Genes that encode molecules expressed on the surfaces of gametes are key to the success of several interactions among males or between males and females, including sperm chemoattraction toward the egg, gamete physiological activation (including the sperm acrosome reaction), sperm binding to the egg coat, and fusion of gametes [26, 52]. Such genes are among the most rapidly evolving parts of animal genomes [34, 80], in part because the gene products are subject to both natural selection associated with fertilization success and sexual selection associated with sperm competition among males or reproductive conflicts of interest between males and females. A frequent outcome of such selection within species is the rapid divergence of protein-coding sequences between closely related species, in part via high relative rates of nonsynonymous nucleotide substitutions that affect the specificity of protein interactions during fertilization [19, 68, 69]. Codon models of nucleotide evolution can be used to identify episodes of diversifying or positive selection associated with specific lineages or specific codons in alignments of protein-coding sequences [4, 5].

Among mammals, considerable research has focused on genes that encode glycoproteins involved in sperm–egg binding. The mammalian egg coat proteins include two members of the ZP gene family (*Zp2* and *Zp3*) that bind sperm in a selective or species-specific manner (reviewed by [6, 38, 77, 78]). Pairs of ZP2 and ZP3 proteins form heterodimers in antiparallel orientation, with the heterodimers joined to ZP1 polymers that appear to have a structural role in forming the zona pellucida [14, 15, 23, 39, 43]. Several studies have documented high rates of molecular evolution of *Zp2* and *Zp3* among closely related mammal species, including episodes of diversifying or positive selection on codons in the known sperm-binding domains of rodent genes [66, 67, 69, 72, 73], and population genetic analyses indicate selection on *ZP2* and *ZP3* in humans ([24, 58]; for counterexamples see [2, 13, 41]).

The identification of the sperm protein(s) responsible for the specificity or selectivity of sperm binding to the egg coat via interactions with ZP2 and ZP3 has been highly contentious (reviewed by [51, 52]). One well-studied candidate gene that was originally identified in mice is the sperm receptor for the zona pellucida called *Zp3r* (also called sperm protein 56 or *Sp56*; [76]). In the mouse genome, *Zp3r* occurs on chromosome 1 in a cluster of protein-coding genes called the regulator of complement activation (RCA; [28]). The mammalian RCA cluster includes two genes that encode the alpha and beta subunits of the C4b-binding protein (*C4bpa*, *C4bpb*); both proteins are expressed in plasma and (like many other genes in the RCA cluster) function in the innate immune system [53]. In rodents, these three genes (*Zp3r*, *C4bpa*, *C4bpb*) occur in tandem, and each encodes a series of 3–8 repeated sushi domains (also known as complement control protein or CCP domains). The sushi domains contribute to the formation of folded monomers that associate into functional multimeric proteins via oligomerization of their C-terminal sequences [27, 53]. Functional ZP3R in the sperm acrosome consists of an oligomer of six or more monomers [10], similar to the organization of C4b-binding protein in plasma. The co-occurrence of the three genes together in the RCA cluster, their similar protein-coding domains, and their similar organization into functional protein oligomers, suggest that they are descended from a common ancestor within the RCA cluster by a series of gene duplication events (e.g., [35]). In contrast to the innate immune function of its paralogs *C4bpa* and *C4bpb*, functional studies show that mouse ZP3R protein binds ZP3 in the egg coat in a species-specific fashion [9, 10, 76]. However, mouse knockout studies that show *Zp3r*-null homozygote males are fertile [47, 52] suggest that ZP3R is not essential for gamete binding. One interpretation of those results is that multiple sperm proteins (including ZP3R) contribute to (and have redundant functions in) sperm binding to the zona pellucida.

Two previous studies focused on selection associated with ZP3R-dependent gamete binding, but both analyses misidentified the gene [44, 45, 58]. The RCA cluster in human and other primate genomes includes only two paralogous gene copies that encode sushi domains (*C4BPA* and *C4BPB*), and does not include a third gene that is orthologous with the rodent gene *Zp3r*. Instead, *Zp3r* is unique to Glires (the rodents and lagomorphs), and descended from *C4bpa* by a gene duplication event in the common ancestor leading to mice, rabbits, and their extant relatives [41]. Consequently, it is clear that Rohlfs et al. [58] documented strong but unexpected evidence for LD between the human gene *C4BPA* in the RCA cluster and the egg coat gene *ZP3*. This evidence is unexpected because it implies that human *C4BPA* is expressed in sperm and mediates gamete binding, which is not a known function or mode of expression for human *C4BPA*. Other complementary evidence has extended that hypothesis to include human *ZP2* coevolution with *C4BPA*, identified a key codon under selection in all three genes, and showed that covariation of pairs of alleles among those genes has a detectable influence on human fertility [24]. Cagliani et al. [11] found many positively selected codons in their analysis of primate *C4BPA*, which they ascribed to the immunological (rather than the reproductive) function of that gene. By contrast, the evidence for *Zp3r* expression and function in the sperm acrosomal vesicle of rodents is clear and well documented, but a codon model analysis of the molecular evolution of *Zp3r* could only have been applied to an alignment of *Zp3r* orthologs from Glires (in which this gene occurs next to *C4bpa* within the RCA cluster), and not outside of that clade. Such an analysis appears not to have been carried out.

Here we analyze the evolution of *Zp3r* and its paralogs in the RCA gene cluster in Glires and in Primates. We used codon models to identify episodes of positive selection on lineages or codons in alignments of RCA genes that are known or suspected to be involved in sperm–egg binding (*Zp3r* in Glires, *C4BPA* in Primates) and alignments of genes that encode sperm receptors in the egg coat (*Zp2*, *Zp3*). As a negative control for the contribution of other modes of selection to the evolution of those genes, we contrasted those codon model results against evidence for positive selection on two paralogous genes in the RCA cluster (*C4bpb* in both taxa; *C4bpa* in Glires) that are not known to be expressed in gametes or sensitive to sexual selection at fertilization, and two genes that encode structural proteins in the zona pellucida (*Zp1*, *Zp4*). As a positive control, we compared those results to models of episodic diversifying selection acting on two genes (*Izumo1*, *Juno*) that are known to be required for sperm–egg fusion and are expected to be sensitive to sexual selection at fertilization [22]. We use these comparisons among genes and taxa to test the working hypothesis that sexual selection on these interacting gene products causes high relative rates of nonsynonymous substitution differences among species.

We found some genes involved in sperm–egg binding in Glires or Primates showed episodes of positive selection, but there was little evidence for more episodes of positive selection on such genes in comparison to the other taxon (in which the same orthologous gene is not know to be expressed in gametes) or in comparison to other genes (that are not involved in gamete binding or gamete fusion). We found some modest evidence for coevolution between sperm- and egg-expressed genes that encode interacting gene products, but this evidence was limited to one specific primate lineage. We offer some speculative interpretation of those surprising (and mostly negative) results, and propose some guidelines for future analyses of these or other genes that mediate gamete interactions under sexual selection.

## Results

### Episodic diversifying selection on RCA genes *Zp3r*, *C4bpa* and *C4bpb*

**Fig. 1:**
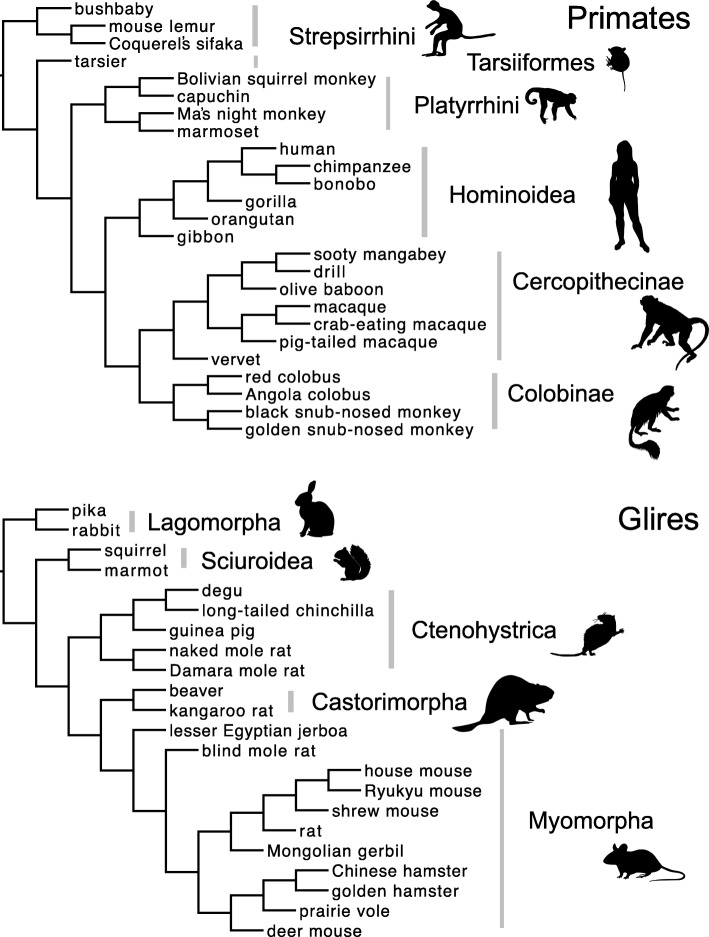
Species trees used in the codon model analyses, including common names for species and higher taxon names for clades, from Springer et al. [65] and Fabre et al. [16]. Organism icons from phylopic.org

**Fig. 2:**
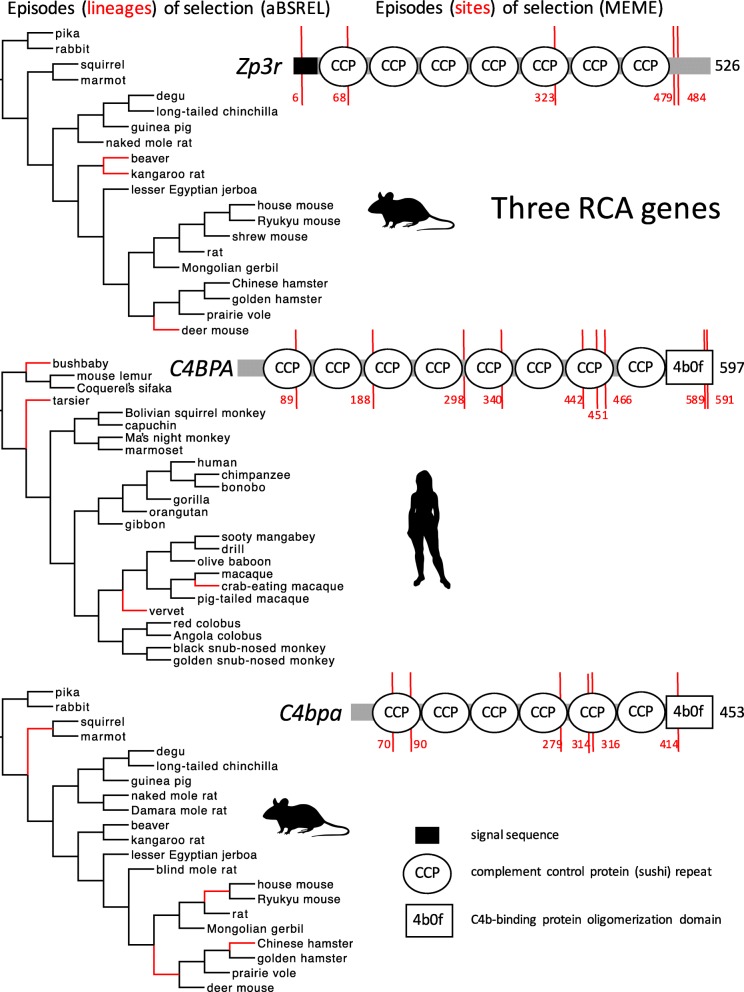
Episodes of diversifying positive selection on three genes from the RCA cluster, including *Zp3r* and *C4bpa* (in Glires), and *C4BPA* (in Primates). *Zp3r* and *C4BPA* are known or expected to be expressed in gametes and sensitive to sexual selection, but *C4bpa* is not. Red branches in each phylogeny show lineages under positive selection in aBSREL analyses. Numbered red vertical bars in each gene cartoon show the locations of codons under positive selection in MEME analyses relative to the total length of the alignment (the grey bar), and relative to several different protein coding domain types. No episodes of diversifying selection were found in *C4bpb* (in Glires) or *C4BPB* (in Primates; not shown)

We used the adaptive branch-site random effects likelihood (aBSREL) model to identify episodes of selection associated with specific lineages (or times in the evolutionary history of the organisms; Fig. 1), and to test the hypothesis of more episodes of positive selection in genes that encode proteins involved in gamete binding or fusion. We found 11 episodes of selection acting on the two RCA genes expressed in sperm, including three episodes of positive selection on *Zp3r* in the deer mouse and both species of Castorimorpha (beaver, kangaroo rat), and four episodes of positive selection on *C4BPA* in the bush baby, tarsier, and two Old World monkeys (vervet, crab-eating macaque) (Table 1; Fig. 2). Episodes of positive selection included about 8% of the total branch length in the phylogeny for each of those genes and taxa, and a mean of about 8% of codons in each alignment were included in the class of codons under positive selection on those branches (Table 1; see Additional file 1: Appendix 1 for a complete summary of the codon model results). By contrast, we found no episodes of positive selection acting on *C4bpb* in Glires or *C4BPB* in Primates (Table 1).

**Table 1:** Summary of codon model results using the aBSREL and MEME methods

gene	taxon	aBSREL				MEME			
		number of selected branches (P < 0.01)	total branches	ω^a^	proportion of codons under selection	number of selected codons (P < 0.01)	total codons	β^b^	proportion of gene tree under selection
*Zp3r*	Glires	3	37	37	0.08	5	527	63	0.20
*C4bpa*	Glires	4	39	293	0.05	6	457	34	0.15
	Primates	4	45	431	0.08	9	597	149	0.11
*C4bpb*	Glires	0	35	n/a	n/a	0	247	n/a	n/a
	Primates	0	47	n/a	n/a	0	256	n/a	n/a
*Zp1*	Glires	3	31	2232	0.04	3	615	181	0.12
	Primates	2	39	10000	0.02	3	634	1436	0.04
*Zp2*	Glires	3	35	3561	0.03	4	589	27	0.22
	Primates	1	47	10	0.08	0	745	n/a	n/a
*Zp3*	Glires	1	37	169	0.04	1	421	7	0.36
	Primates	0	47	n/a	n/a	0	424	n/a	n/a
*Zp4*	Glires	1	37	10000	0.02	2	532	114	0.11
	Primates	2	47	6010	0.02	3	541	188	0.04
*Izumo1*	Glires	2	41	51	0.15	1	262	111	0.10
	Primates	1	45	10000	0.05	0	350	n/a	n/a
*Juno*	Glires	0	41	n/a	n/a	1	243	20	0.13
	Primates	0	41	n/a	n/a	0	250	n/a	n/a

For each analysis (one model fitted to one gene from one taxon) the primary response variable is shown on the left (the number of episodes of positive selection), followed by the size of the sample (the total number of branches in the gene tree or codons in the alignment) and the two secondary response variables (the mean value of the model parameter for episodes of positive selection, and the mean proportion of codons or branch lengths with that model parameter value)

^a^ Mean estimated value of dN/dS at some codons along positively selected branches; values of 10,000 are high but imprecisely estimated (at the boundary condition for the parameter value)

^b^ Mean estimated value of dN along some positively selected branches at positively selected codons

Those results, especially the difference between genes involved in fertilization (*Zp3r*, *C4BPA*) compared to a gene involved only in innate immunity (*C4bpb*), might be interpreted as evidence pointing toward specific primates, rodents, or lagomorphs that have experienced sexual selection associated with the specificity of sperm–egg binding at fertilization. However, other comparisons between genes and taxa did not support that interpretation. Specifically, we found four episodes of positive selection on *C4bpa* in Glires including the Chinese hamster, the internal branch leading to the most recent common ancestor of squirrel and marmot, the internal branch leading to the most recent common ancestor of two species of *Mus*, and the lineage that includes the most recent common ancestor of hamsters, voles, and deer mice (Fig. 2). Like the results for *Zp3r* in Glires and *C4BPA* in Primates, the relative rate of nonsynonymous substitution was high (ω = 15–693, mean = 293) at positively selected codons along those four branches of *C4bpa* in Glires, and a substantial proportion (1–9%, mean = 5%) of codons in the *C4bpa* alignment was included in that positively selected class (Table 1).

Because *C4bpa* in Glires is not known to be involved in fertilization, those episodes of positive selection cannot be ascribed to sexual selection. The contrast between *Zp3r* evolution (including three episodes of positive selection) and *C4bpa* evolution (four episodes) does not suggest an especially strong effect of sexual selection on *Zp3r* in Glires. Similarly, because *C4BPA* is known to be involved in innate immunity in Primates and is suspected to be involved in fertilization in humans and perhaps in other Primates, the contribution of sexual selection to *C4BPA* evolution is expected to be evident as more episodes of positive selection on *C4BPA* in Primates compared to *C4bpa* in Glires. However, we found the same number of episodes of positive selection on *C4BPA* in Primates and on *C4bpa* in Glires. Thus our planned comparisons between genes (in Glires) or between taxa (for *C4bpa*) do not support the hypothesized role for sexual selection in the evolution of RCA genes.

We used the mixed effects model of evolution (MEME) to identify positive selection associated with specific codons as a complementary way to test the hypothesis of more episodes of positive selection in genes that encode proteins involved in gamete binding or fusion. We found little evidence for a strong contribution of sexual selection toward the number of episodes (codons) of selection on RCA genes. We found five codons under selection in *Zp3r* codons in Glires and nine codons under selection in *C4BPA* in Primates (Fig. 2), which represented 0.9–1.5% of codons in each gene alignment (Table 1). Similar to the aBSREL analyses, we found no codons under selection in *C4bpb* in Glires or *C4BPB* in Primates (Table 1).

Like the aBSREL results, comparisons of MEME models between genes for a single taxon (*Zp3r* versus *C4bpa* in Glires) and between taxa for a single orthologous gene (*C4BPA* in Primates versus *C4bpa* in Glires) did not strongly support the hypothesis of more episodes of positive selection in genes that encode proteins involved in gamete binding. We found six codons under selection in the alignment of *C4bpa* in Glires compared to five codons under selection in *Zp3r* (five) (Fig. 2). That comparison between MEME results does not suggest an especially strong effect of sexual selection on the evolution of *Zp3r* (in fertilization) compared to the effect of natural selection on *C4bpa* (in innate immunity) among Glires. Only one of our MEME model comparisons supported the hypothesized effect of sexual selection on episodes of positive selection: we found slightly more codons (nine) under selection in *C4BPA* in Primates (involved in both fertilization and innate immunity) compared to the number of codons (six) under selection in *C4bpa* in Glires (involved in innate immunity alone) (Fig. 2; Table 1).

### Episodic diversifying selection on four egg coat genes

**Fig. 3:**
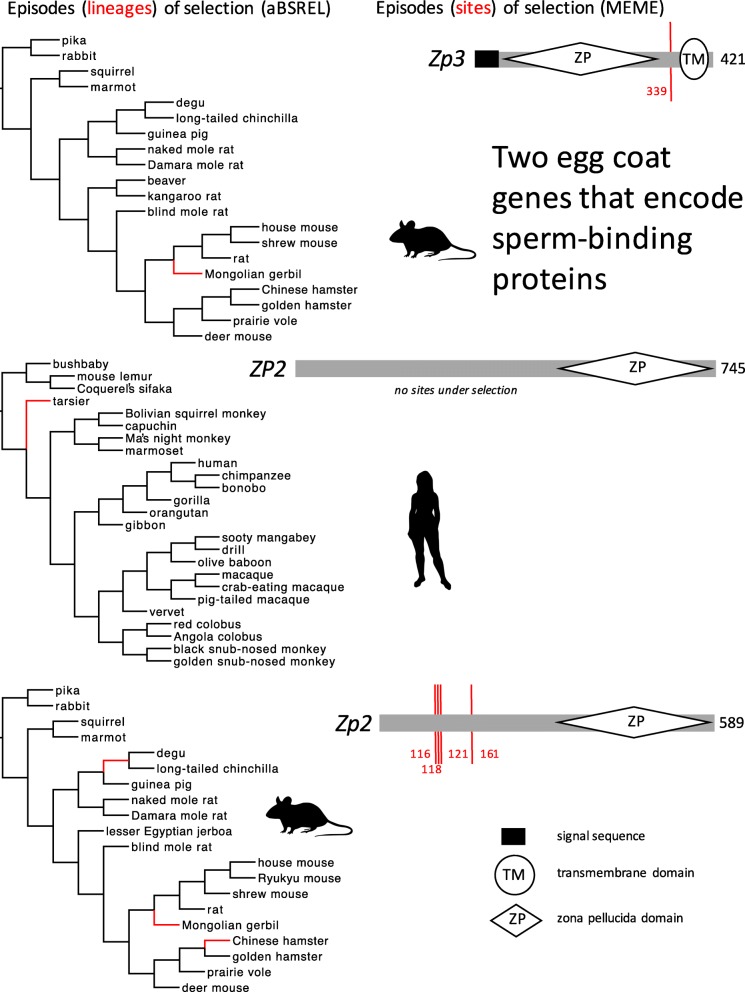
Episodes of diversifying positive selection on two genes that encode egg coat proteins that bind sperm, including *Zp3* and *Zp2* (in Glires), and *ZP2* (in Primates). Both genes in both taxa are known to be expressed in the egg coat and sensitive to sexual selection. Note that no positively selected sites were identified in *ZP2* in Primates. Branches and sites under positive selection, and protein coding domain types, are shown as in Fig. 2. No episodes of diversifying selection were found in *ZP3* in Primates (not shown)

**Fig. 4:**
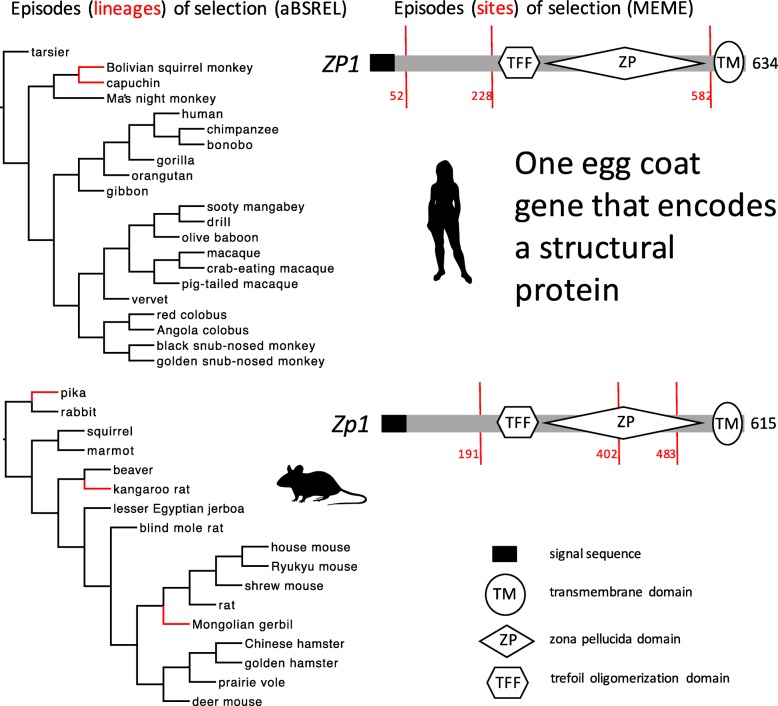
Episodes of diversifying positive selection on an egg coat structural gene (*Zp1*) that does not bind sperm and is not expected to be sensitive to sexual selection. Branches and sites under positive selection, and protein coding domain types, are shown as in Fig. 2

**Fig. 5:**
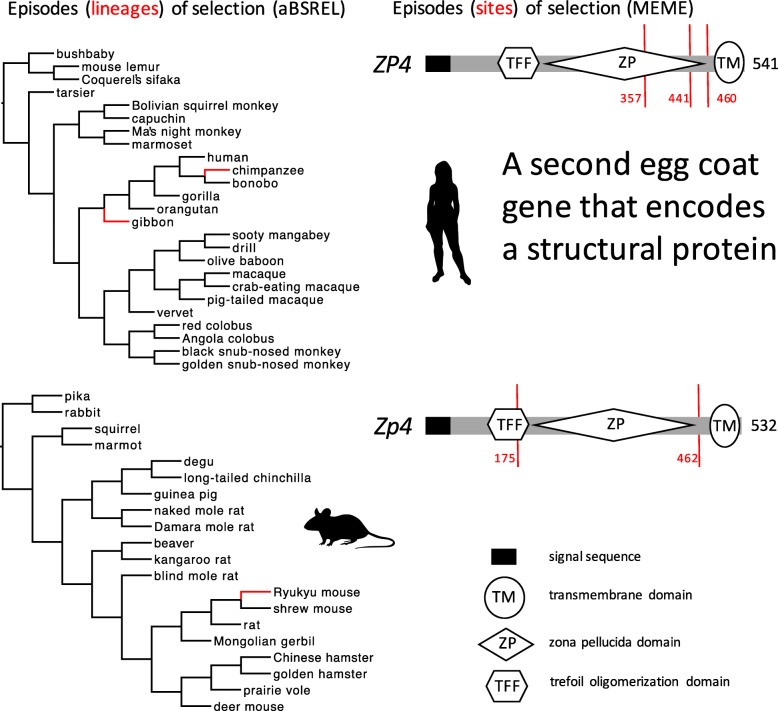
Episodes of diversifying positive selection on a second egg coat structural gene (*Zp4*) that does not bind sperm and is not expected to be sensitive to sexual selection. Branches and sites under positive selection, and protein coding domain types, are shown as in Fig. 2

In aBSREL models, we found 1–3 episodes of positive selection on *Zp2* or *Zp3* in Glires and *ZP2* in Primates (Fig. 3), but we found no episodes of positive selection on *ZP3* in Primates (Table 1). We found comparable numbers of episodes (1–3) of positive selection for all alignments of *Zp1* (Fig. 4) and *Zp4* (Fig. 5) in Glires or *ZP1* and *ZP4* in Primates. The total number of episodes of positive selection (eight) for these two orthologs that do not encode selective or specific sperm-binding molecules (*Zp1, Zp4*) was greater than the total number of episodes of positive selection (five) for the two orthologs (*Zp2, Zp3*) that are known to play a role in selective or specific sperm binding. In MEME models, we found 1–4 episodes of positive selection at some *Zp2* or *Zp3* codons in Glires (Fig. 3), but we found no episodes of positive selection at any *ZP2* or *ZP3* codons in Primates. Like the aBSREL analyses, we found comparable numbers of episodes (codons) of positive selection (2–3) for all four alignments of *Zp1* (Fig. 4) and *Zp4* (Fig. 5) in Glires or *ZP1* and *ZP4* in Primates, and the total number of episodes of positive selection (11 codons) was greater for those two orthologs that do not encode sperm-binding molecules (*Zp1, Zp4*) in comparison to the total number of episodes of positive selection (five codons) for two orthologs that encode selective sperm-binding molecules (*Zp2, Zp3*) (Table 1). The discovery of many positively selected codons (on some lineages) in *Zp1* is particularly strong evidence against the sexual selection hypothesis because these were the smallest alignments that we analyzed (only 17 species in Glires, and only 22 species in Primates), with fewer lineages in each of those gene trees on which to model rate variation among codons. In spite of that constraint, we found more codons (and more lineages) under positive selection for those two structural genes than for two genes known to encode sperm-binding proteins.

### Episodic diversifying selection on two sperm–egg fusion genes

**Fig. 6:**
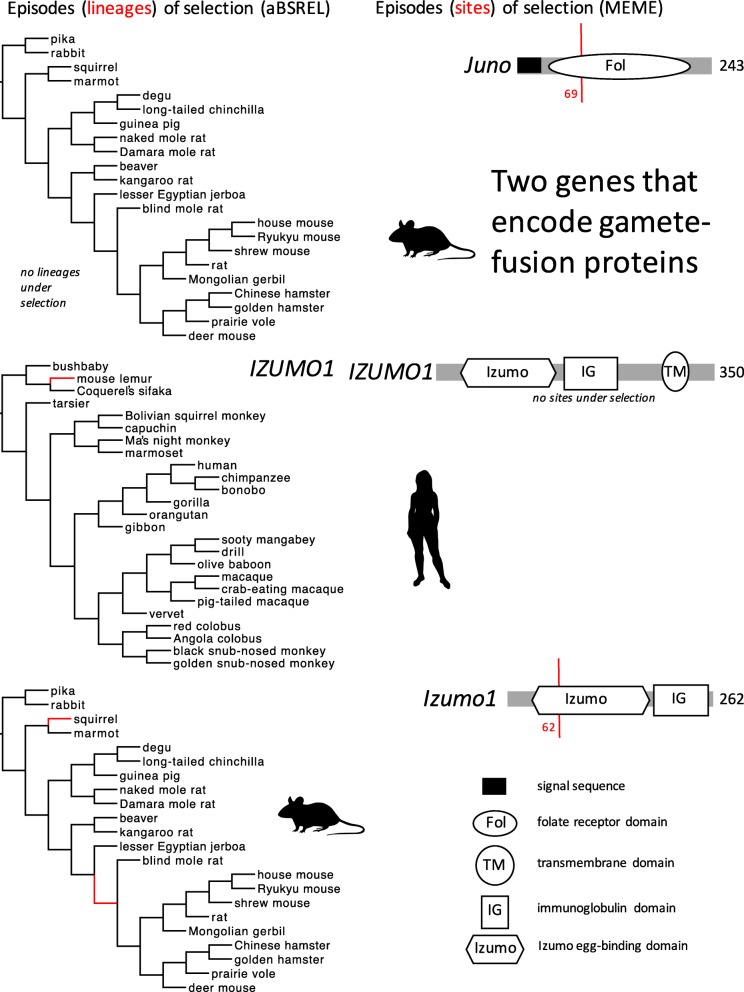
Episodes of diversifying positive selection on three genes that encode gamete-fusion proteins, including *Juno* and *Izumo1* (in Glires), and *IZUMO1* (in Primates). Both genes are known or expected to be expressed in gametes and sensitive to sexual selection. Note that no positively selected lineages could be identified in *Juno* (in Glires), and no positively selected sites could be identified in *IZUMO1* (in Primates). Branches and sites under positive selection, and protein coding domain types, are shown as in Fig. 2. No episodes of diversifying selection were found in *JUNO* in Primates (not shown)

In contrast to the evidence noted above for episodes of diversifying selection on lineages or codons in two suites of genes that encode sperm–egg binding molecules (including some molecules that are also expressed in innate immunity), we found very little evidence for episodes of selection acting on two gamete fusion genes. In aBSREL models, we found one or two episodes of positive selection on *Izumo1* expressed in sperm of Glires or *IZUMO1* expressed in sperm of Primates (Fig. 6), but no lineages under selection in either taxon for the gene (*Juno*) that encodes the cognate molecule that is expressed in eggs and binds IZUMO1 on sperm. In MEME models, we found just one episode of positive selection in *Izumo1* and one episode of positive selection in *Juno* (in Glires) (Fig. 6), but no episodes of positive selection in either gene in Primates (Table 1). The absence of consistent evidence for episodes of selection in comparisons between the two gene pairs (in aBSREL results) and the absence of consistent evidence for episodes of selection in comparisons between the two taxa (in MEME results) did not support the predicted effect of sexual selection on the evolution of genes that encode gamete fusion molecules. Those results are broadly similar to the analyses by Grayson [22] using many of the same sequences but a different model of codon evolution, in which the evidence for positively selected codons in *Juno* and *Izumo1* was limited to other mammal lineages and was weak or absent in Glires and Primates.

### Coevolution between genes that encode interacting gene products

We used the branch-site unrestricted statistical test for episodic diversification (BUSTED) to predict positive selection in an egg coat gene based on observed episodes of positive selection in a sperm-expressed gene. We found only one case of apparent coevolution: between *ZP2* and *C4BPA* in Primates. Primate *C4BPA* evolution included four episodes of positive selection (in the bush baby, tarsier, crab-eating macaque, and vervet lineages; Fig. 2); when we fitted nested codon models of *ZP2* evolution with those four lineages in the foreground class and two or three classes of codons with different substitution rates, we obtained a significantly better model fit by the likelihood ratio test [2δln(L) = 13.2, *p* = 0.00028] for the unconstrained model that included a third class of positively-selected *ZP2* codons. That better model included about 7% of codons in the *ZP2* alignment with a high relative rate of nonsynonymous substitution (ω = 6.2) along those four foreground branches. The specific source of that signal of coevolution between *C4BPA* and *ZP2* was evident from comparing the aBSREL model results for those two genes: both aBSREL results included an episode of positive selection at some codons along the terminal branch leading to the tarsier (Figs. 2, 3). When we dropped the tarsier lineage from the foreground class in the BUSTED model analysis of *ZP2*, we obtained a nonsignificant improvement in the model fit [2δln(L) = 1.0, *p* = 0.59] for the unconstrained model, which suggested no coevolution between *ZP2* and *C4BPA* along those other three branches of the species tree.

No other BUSTED analyses suggested evidence of coevolution between sperm- and egg-expressed gene pairs that encode interacting gene products. Nested models of *ZP3* evolution in Primates did not indicate coevolution with *C4BPA* along the branches of the *C4BPA* gene tree that showed episodes of diversifying positive selection in Primates (Fig. 2). No models of *Zp2* or *Zp3* evolution in Glires indicated that either of those genes coevolve with *Zp3r*. And neither of the BUSTED analyses indicated coevolution between *Izumo1* and *Juno* in Glires or between *IZUMO1* and *JUNO* in Primates.

### Exploratory analysis of functional or phenotypic associations with episodes of selection

We did not preregister hypothesis tests about which specific codons in these gene alignments, or which specific lineages in the phylogenies for Glires and for Primates, are expected to be associated with episodes of diversifying positive selection. Here we explore several possible associations that were suggested by our results but not analyzed in hypothesis tests.

The Philippine tarsier (*Carlito syrichta*) was the only primate lineage associated with multiple episodes of diversifying positive selection in two genes that encode interacting gene products involved in fertilization (*C4BPA* and *ZP2*) (Figs. 2, 3). Such episodes of selection could be caused by life history traits that are associated with especially strong competition among males or among sperm, or by conflicts of interest between mates, and could lead to coevolution of the male- and female-expressed genes during coincident episodes of selection. However, the mating systems of tarsiers do not include traits that are usually associated with sperm competition or sexual conflicts of interest in primates. Males are slightly (about 14%) larger than females in *C. syrichta* [32], individual home ranges overlap slightly, and social groups typically consist of one adult male and one or two adult females plus offspring [50]. In comparison to other primate mating systems that feature pronounced male-biased sexual size dimorphism (e.g., gorilla; [36]), or coercive mating with conflict between the sexes (e.g., chimpanzees; [46]), tarsiers seem to be unlikely hotspots for episodic diversifying selection on fertilization genes. One interpretation of our discovery of coevolution between *C4BPA* and *ZP2* in *C. syrichta* is that this discovery points toward previously unsuspected strong sexual selection in this lineage, but an alternative interpretation is that our discovery is a false positive (or at least unrelated to the mating system of tarsiers). A follow-up study specifically directed at testing those interpretations is needed.

Other examples of multiple episodes of diversifying positive selection in one lineage seem to argue more strongly against the hypothesis that these sperm- and egg-expressed genes coevolve under selection driven by the interaction of the gene products at fertilization. We found multiple episodes of diversifying positive selection in the kangaroo rat (*Dipodomys ordii*) including the sperm acrosomal gene *Zp3r* and the egg coat gene *Zp1*, but those two gene products are not known to interact at fertilization; instead, ZP1 is thought to form the structural component of the fibrillar protein network in the rodent egg coat. Similarly, we found multiple episodes of diversifying positive selection in the Chinese hamster (*Cricetulus griseus*) in the sperm-binding gene *Zp2* and in *C4bpa*, but that RCA gene is not known to be expressed in sperm (or involved in fertilization) in rodents; instead, *C4bpa* is thought to function only in the innate immune system of hamsters and other Glires. Finally, we found multiple episodes of diversifying positive selection in the Mongolian gerbil (*Meriones unguiculatus*), including the three zona pellucida genes *Zp1*, *Zp2*, and *Zp3*, but not including *Zp3r* or other sperm-expressed genes; those episodes of selection on all three egg coat genes might point to interesting coevolution among those genes (and interactions among their gene products to form the gerbil egg coat), but they do not seem to point to the effects of sexual selection on molecular evolution.

The discovery of some codons under selection in egg coat genes could potentially be related to the known function of specific domains in those genes. The only positively selected codon that we found in Glires *Zp3* (codon 339 in our trimmed *Zp3* alignment; Fig. 3; Additional file 2: Appendix 2) occurred in the portion of the gene that encodes the known sperm-binding site (sometimes called the sperm-combining site), between the ZP domain and the transmembrane region near the carboxyl end of the mature protein. That discovery alone would be consistent with the predicted effects of sexual selection on the evolution of selective sperm binding by the egg coat, and is consistent with previous documentation of high rates of nonsynonymous substitution in the ZP3 sperm-binding site in other analyses of *Zp3* evolution in rodents (e.g., [72, 73]). However, other results were not consistent with those predicted effects. First, we found a larger number of positively selected codons (four) in Glires *Zp2* (codons 116, 118, 121, 161 in our *Zp2* alignment; Additional file 2: Appendix 2) but all of those codons occurred outside of the N-terminal region of ZP2 that both confers specificity of sperm binding in mice [7] and covaries with fertility in humans [24]. Second, we found no positively selected codons in *ZP2* or *ZP3* of Primates inside or outside of the known sperm-binding sites in those genes, and we found no positively selected codons in *Juno* from either taxon, including sites inside or outside of the parts of each gene that encode the regions known to mediate protein-protein interactions involved in fertilization. Those additional results seem to considerably weaken the overall strength of evidence for diversifying positive selection specifically on the sperm-binding domains of these genes.

It is less straightforward to assign possible functional significance to codons under positive selection in alignments of RCA genes because substrate binding by those multimeric proteins depends on the number and organization of the monomers (and their possible interaction with a beta subunit protein in the oligomer), and in particular because the egg-binding function of those gene products (ZP3R in rodent sperm, C4BPA in human sperm) has not been studied. Indirect insight into possible functional associations could be based on comparisons between paralogous genes with different functions in the same taxon (*Zp3r* versus *C4bpa*) or between taxa in which the same ortholog is thought to differ in function (*C4bpa* versus *C4BPA*).

## Discussion

We found only limited evidence for diversifying positive selection associated with the gamete-binding function of fertilization genes in both Glires and Primates. We expected to find more episodes of positive selection in a gene with multiple functions including innate immunity and sperm-binding to the egg coat (*C4BPA* in Primates) compared to the same orthologous gene without a role in fertilization in the other taxon (*C4bpa* in Glires), and we expected to find more episodes of positive selection in a second gene with a known role in gamete-binding (*Zp3r* in Glires) compared to a paralogous gene without a fertilization function in the same taxon (*C4bpa*). Neither of those predictions was supported by the model results. Comparable patterns for genes expressed in the zona pellucida, and for two genes that mediate sperm–egg fusion, reinforced this apparent lack of evidence for many episodes of diversifying positive selection associated with two different modes of sperm–egg interaction. We conclude that these data offer little support for the hypothesis that sexual selection shapes the molecular evolution of those gene products in these two taxa at this taxonomic level of comparison (within crown group taxa that are each about 70 Ma old). Similar comparative approaches that contrasted genes with and without a function in fertilization in the same taxon (e.g., [71]), and approaches that contrast homologous genes with different function or expression patterns in different taxa (e.g., [74]), have provided important insights into the causes of selection at the molecular level, and the processes that mediate the response to such selection.

Our study and discoveries benefited from many of the advantages that have been proposed for preregistration as an approach to avoid false positives in evolutionary ecology [17] and other disciplines [62], such as the selective reporting of some model results at the expense of others (sometimes called cherry-picking) or the development of open-ended post hoc hypotheses after the results are known (sometimes called HARKing). Codon model analyses of positive selection seem particularly susceptible to the allure of these questionable research practices because the models can be fitted to data without specifying particular species or coding sequence domains that are expected to be the targets of selection. Constraining our analysis and reporting to include all results (and not just those results that might have conformed to our broadly stated expectations) may help to avoid selective reporting of some results, and seems more likely to lead to an unbiased view of the magnitude and targets of selection.

Our study did not address an alternative working hypothesis: that the response to sexual selection acting on fertilization genes may be mediated by the evolution of gene expression differences rather than by the evolution of substitution differences. Mammal species show substantial qualitative differences in the expression of RCA genes and egg coat genes, including the gain of expression of a new paralog (*Zp3r*) in Glires, and the loss of expression of some ZP family genes as pseudogenes in diverse mammal lineages including the loss of *Zp4* expression in mice [21, 41]. The evolution of those qualitative expression differences suggests that other quantitative differences in expression might also mediate responses to sexual selection acting at fertilization. The observation that the gain and loss of gene function has not included *Zp2* and *Zp3* suggests that the evolution of quantitative expression differences might be constrained by functional requirements for specific gamete-binding functions (such as essential binding sites in both ZP2 and ZP3 protein subunits). However, there might be considerable scope for selection to modulate relative expression levels within the egg coat or within the sperm acrosome. Promoter regions associated with *Zp2* and *Zp3* coding sequences are known (e.g., [42]), and are reported to be highly conserved between Primates and Glires (e.g., [37]). Analyses of the evolution and functional variation of these regulatory sequences or of the genes that encode their cognate regulatory molecules (such as repressors) might reveal evidence of responses to sexual selection that were not evident in our analyses of coding sequence evolution.

Our results raise at least two additional questions. First, given the existence of other strong evidence for positive selection acting on genes that mediate fertilization interactions and are sensitive to sexual selection, what is the appropriate genomic scale for this comparative approach? Here we focused on a small number of paralogous CCP-containing genes in the RCA cluster plus a small number of genes from a second gene family (ZP-domain genes), including some pairs of genes in those two gene families that are known or expected to encode interacting gene products. We found that sperm-expressed genes (e.g., *Zp3r* in Glires) and egg-expressed genes (e.g., *Zp2*, *Zp3*) had not experienced more episodes of positive selection in comparisons that were restricted to the most closely-related (and in some ways most directly comparable) parts of the same genomes (*C4bpa*, *C4bpb*; *Zp1*, *Zp4*). However, it is possible that a broader comparison across the genomes or across the gonad transcriptomes of Glires and Primates might show that these few fertilization genes fall in the far tail of the frequency distribution of strongly positively selected genes. The interpretation of such patterns involving positive selection detected across the genome (e.g., [59, 75]) is complicated by the diverse nature of the structure and function of the genes in the comparison, and by the expectation that they are subject to diverse modes and sources of selection. We find this type of focused comparison (e.g., [25]), limited to a few other genes in the same gene family (ZP-domain) or in the same genomic region (CCP-containing genes), or with a similar function in fertilization (*Izumo1*, *Juno*), to be highly informative because such comparisons focus on genes that are expected to have similar functional properties and experience comparable modes of selection. Focused comparisons among such genes seem to have the greatest potential to reveal differences in the episodic nature of selection on genes that are or are not expressed in gametes and subject to sexual selection at fertilization. A broader genomic comparison might lead to different insights into the relative importance of the few episodes of positive selection in gamete-recognition genes that were identified in our analyses.

Second, what is the appropriate temporal or phylogenetic scale for comparative analyses of gamete-recognition genes among taxa? Increased taxon sampling improves the scope for identifying some lineages under positive selection (at some codons in aBSREL models), and improves the scope for identifying some codons under positive selection (along some lineages in MEME models). However, broader comparisons among more distantly-related taxa can be confounded by gap-filled alignments due to the accumulation of real insertion-deletion mutations and due to the accumulation of multiple substitutions that lead to convergent similarities or dubious alignment among highly divergent gene copies from distantly related lineages. Both of those constraints will cause multiple sequence alignment algorithms to infer gap-filled alignments with reduced power to detect positive selection, and may cause misaligned codons to differ at nonsynonymous nucleotide sites (leading to false positives in codon model results). Previous analyses of some of the genes analyzed here found strong evidence of positively selected codons that encode sperm-binding sites in zona pellucida genes of mice, but only in analyses focused on congeneric species [72, 73]. In their analysis of the molecular evolution of complement genes among a diverse suite of Primates, Cagliani et al. [11] found 15 codons under selection in C4BPA (using different criteria from those used in our study to identify positively selected codons), but this was not an unusual proportion of codons under selection (15%) in comparison to other complement genes in their analyses (range 5–35% among 18 genes that showed evidence of positive selection). Our previous analyses of positive selection on gamete-recognition genes from diverging populations or congeneric species of sea stars [25, 55] used similar combinations of phylogenetic and population genetic approaches to identify codons under selection that may be associated with variation in fertility or gamete compatibility. Possibly the evidence for selection acting on such genes is more likely to be detected when sampling focuses on relatively recent episodes of selection. Planned comparisons of codon model results for fertilization genes sampled on increasingly broad phylogenetic scales (e.g., [1]) are needed to test that possibility.

## Conclusions

Codon model analyses of protein-coding sequences provide a powerful method for testing hypotheses of selection acting on codons or lineages associated with specific functional features of genes and organisms. A comparative approach that contrasts taxa with different phenotypic traits or contrasts genes with different functional expression patterns can provide important context for interpreting codon model results. We found both codons and lineages under episodic diversifying selection among mammalian species in two clades in which different RCA genes have been implicated in sperm–egg interactions, and those results alone could be interpreted as evidence for sexual selection associated with variation in fertilization success. However, comparisons of codon model results between paralogous genes (with and without a function in fertilization) and between orthologous genes (in taxa with different expression patterns) did not support that interpretation. We conclude that caution is warranted in ascribing any of those particular results to the effects of sexual selection. We advocate for preregistration of analyses and interpretations in future studies, including comparative analyses of molecular evolution among genes and among taxa that can be used to test a specific hypothesis about the causes of selection acting on molecules and organisms.

## Methods

### Comparative analysis of genes and taxa

We used a common comparative approach to analyze and interpret evidence for diversifying positive selection in codon models caused by sexual selection acting on some genes that encode sperm–egg binding proteins (ZP3R, C4BPA; ZP2, ZP3) or gamete fusion proteins (IZUMO1, JUNO) in some taxa. We compared those results to the same models fitted to alignments for paralogous genes in the same taxon (*C4bpa*, *C4bpb*, *C4BPB*; *Zp1*, *Zp4*) that are not sensitive to sexual selection, or to the same models fitted to an alignment for the orthologous gene in the other taxon (*C4bpa*) in which the gene is not expressed in gametes. We also looked for coevolution between sperm- and egg-expressed genes by searching for single lineages (internal branches or terminal leaves) in the Glires or Primates phylogeny that showed evidence of positive selection in both members of gene pairs that encode interacting proteins.

This approach is similar to the well-known comparative approach used in previous studies (e.g., [69]). The main advantage of this comparative approach is that it can be used to test a specific working hypothesis: more evidence of diversifying positive selection (more codons or lineages) in genes that are sensitive to sexual selection in comparison to other genes (in the same taxon) or other taxa (for the same genes) that do not mediate gamete interactions and are not sensitive to sexual selection. Codon models can be fitted to sequence alignments without a specific hypothesis about which genes or taxa are expected to show evidence of positive diversifying selection, but this unsupervised mode of analysis is more sensitive to false positives when the model results are interpreted post hoc. Unsupervised use of likelihood ratio tests in codon models has been criticized as likely to generate false positives (e.g., [18, 30]). Several solutions to this problem of unconstrained searches for positive selection have been proposed (e.g., [8, 82]), including the specification of hypotheses based on known differences in expression and function between genes and between taxa, such as the contrast between codon model results for candidate genes under selection in comparison to so-called housekeeping genes (e.g., [3]).

We followed a preregistered protocol of codon model analyses to test hypotheses about the influence of sexual selection on the molecular evolution of RCA genes and other sperm-expressed genes and their egg-expressed cognate genes. We preregistered those methods in order to avoid problems associated with the exercise of researcher degrees of freedom in the selection and interpretation of analyses and hypothesis tests (sometimes called the garden of forking paths) [17, 20, 61]. Our preregistered workflow was finalized and deposited in the preregistration database at the Open Science Framework (osf.io/yf9be) before we obtained the sequence data used in our analyses. The preregistration included both our plans for obtaining and handling data and our plans for hypothesis tests. Here we note specific deviations from that workflow that arose during data handling and analysis, including exploratory analyses or tests that were not preplanned.

### Taxon choice, data assembly, and sequence alignment

We used mouse genes (for Glires) or human genes (for Primates) as query sequences to search the Ensembl database (release 91; [81]) for coding sequences of orthologous genes in other sequenced mammalian genomes, including 19 Glires and 24 Primates. We used mouse and human orthologs as queries because most of the available experimental annotation for gene function comes from biochemical or genetic analyses of mouse and human genes. Our analysis focused on alignments for genes from each taxon separately (and not analyses of genes for Glires and Primates together in one alignment) because one key gene (*Zp3r*) is unique to Glires, and our hypothesis testing depended on comparison of results among genes (with different functions in the same taxon) or between taxa (in which one orthologous gene has evolved two different functions). The two taxa are particularly well suited for this kind of comparative approach because they are closely related (and make up the large majority of species in the supertaxon Euarchontoglires), and because the crown group is estimated to be of similar age in each taxon: 71–63 million years ago for Primates [65]; 75–71 million years ago for Glires [64].

We downloaded from Ensembl each 1:1 ortholog that had whole genome alignment coverage (WGA) and gene order conservation (GOC) scores greater than 75. For 1:1 orthologs that included multiple transcripts of different length, we chose the longest isoform. For 1:1 orthologs that failed to pass either of those two filters, we downloaded the Ensembl sequence and confirmed its identity by using the Ensembl sequence as the query in a blastn search against all mouse (or human) sequences in GenBank. For cases in which Ensembl did not identify a 1:1 ortholog of the mouse (or human) gene, or for cases where the 1:1 ortholog with a low WGA or GOC score was not a best blast match to the expected mouse (or human) gene, we used the mouse (or human) ortholog as the query in a blastn search of all GenBank sequences for that species. For those blastn searches we used two search criteria (expectation scores of e < 10^− 40^ and query coverage greater than 75%) to find a GenBank accession that was orthologous to the mouse (or human) gene. Those blastn searches also identified orthologs from four other species (beaver, marmot, Mongolian gerbil from Glires; red colobus from Primates) for which genome assemblies and gene models were available as sequence accessions in GenBank but not searchable in Ensembl. Cases in which Ensembl contained no 1:1 ortholog and blastn searches did not identify a likely ortholog in GenBank were scored as missing; alignments thus varied in size from a maximum of 22 species (Glires) or 25 species (Primates) to a minimum of 17 species (*Zp1*, Glires) or 22 species (*ZP1*, Primates) (see Additional file 3: Appendix 3 for taxon names and accession numbers for each sequence).

We used COBALT [54] to align orthologs within Glires or within Primates. We used the COBALT method because it is sensitive to the organization of protein-coding genes into distinctive functional domains (such as the sushi domains of many RCA genes). We used the default values for COBALT alignment parameters (gap open and extension penalties). Although COBALT successfully conserved the boundaries between coding sequence domains in genes, our preliminary codon model analyses of COBALT alignments included many codons under selection that occurred in parts of the alignments with many gap sites (which may be incorrectly aligned). Alignment errors can cause numerous false positives in codon model analyses [31, 40, 57]. The benefit of removing alignment errors (by deleting gap-filled parts of alignments that may be of dubious homology) is probably greater than the cost of shorter alignments (with fewer sites and reduced power to detect positive selection [56]. For those reasons, we revised the COBALT alignments using two criteria that were not part of our preregistered workflow. First, we examined each alignment for any amino acid sequence motifs for one species that were obviously misaligned with a nearby region of other species (i.e., COBALT errors caused by a high gap opening penalty), and we manually adjusted those regions of each alignment (e.g., a distinctive and obviously misaligned four-codon motif in the 3′ region of *C4BPB* in the tarsier). Second, we examined each alignment for short (≤30 codon) motifs in one sequence that were separated from other parts of the alignment by gaps at both the 5′ and 3′ ends of the motif. We assumed that such islands of codons were likely to represent compressed sequences with many possible alignment errors (e.g., part of *Izumo1* in the kangaroo rat). If more than half of the codons in such islands encoded amino acid differences from other sequences in the alignment, then we recoded those islands of codons as missing (replaced with alignment gaps) to represent uncertain homology with other sequences for that region of the alignment. We then used trimAL v1.2 [12] to delete sites in each alignment that were represented by sequence data for < 80% of species. We used the norMD score [70] to assess overall alignment quality with a cutoff value of 0.6 (all alignments passed that filter).

### Phylogeny selection for lineage specific analysis

Codon models are used to estimate parameter values associated with episodes of positive selection by mapping synonymous and nonsynonymous nucleotide differences onto a phylogeny (Fig. 1). We used the canonical species tree topology and higher taxon names from Springer et al. ([65]; Fig. 1) for Primates. We used the canonical species tree topology and higher taxon names from Fabre et al. ([16]; Fig. 2) for Glires. We edited the Newick string for each species tree to match the species represented in each alignment for each taxon (17–25 species per alignment), collapsed nodes for missing species accordingly, and added that Newick string to each alignment file as input for codon model analyses (see Additional file 2: Appendix 2 for all sequence alignments and input files for codon model analyses).

We also estimated gene trees for each multiple sequence alignment. An initial empirical protein evolutionary model was determined for each alignment using ModelGenerator v.85 [33]. Phylogenetic reconstruction was performed using MrBayes [29] under the best fit empirical protein evolutionary model. Two independent MCMC chains were run for 500,000 generations with print frequency at 1000 and sample frequency set at 10. The consensus tree was estimated following a burnin of 25%. We used the Shimodaira-Hasegawa (SH) test implemented in TreePuzzle [60] to ask whether the canonical species tree was a significantly worse (*p* < 0.05) fit to the data for each alignment in comparison to the best gene tree estimated by MrBayes. In three cases where the SH test indicated a better fit to the data for the best gene tree (*C4BPA* in Primates; *ZP1* in Primates; *Zp2* in Glires), we used both trees in codon model analyses and asked whether our results differed between those two analyses (in all three cases we recovered the same episodes of positive selection that were also identified in analyses using the canonical species tree).

### Codon model analyses

We fitted three models of episodic diversifying selection to coding sequence alignments. We used the MEME method [49] to identify codons in each alignment that were estimated to have high relative rates of nonsynonymous substitution (ω) along some lineages in the species tree. We used the aBSREL method [63] to identify lineages in each species tree that were estimated to have high values of ω for some codons in the alignment. We implemented those models using the datamonkey web interface [79]. In each of those analyses, the primary parameter value was the number of episodes of diversifying or positive selection (codons under selection in the MEME analyses; branches under selection in the aBSREL analyses). We used a relatively stringent criterion (a likelihood ratio test result with *p* < 0.01) to identify those episodes of positive selection from the analysis of each alignment; we chose this lower critical *p* value (compared to the proposed critical p value in our preregistration) because we carried out several hypothesis tests for each alignment (a MEME and an aBSREL analysis, plus one or two additional analyses in some cases; see below) and because we were concerned about possible false positives associated with alignment errors. In each analysis we also noted two secondary parameter values: the proportion of the gene tree under positive selection, and the value of β (the nonsynonymous substitution rate along those branches), for each positively selected codon in MEME models; and the proportion of codons under positive selection, and the value of ω (the relative rate of nonsynonymous substitution at those codons), for each positively selected branch in aBSREL models.

We also used the BUSTED method [48] to characterize the strength of coevolution between pairs of male- and female-expressed genes under selection. This model allows the user to assign branches in the gene tree to a class of foreground lineages based on an a priori hypothesis, and then ask whether alignment-wide evidence of positive selection can be detected as a better fit for a model with a high value of ω at some codons on the foreground branches (relative to a null model without positive selection at some codons on foreground branches). We used the aBSREL results for sperm-expressed genes involved in fertilization (*Zp3r* in Glires; *C4BPA* in Primates; *Izumo1* in each taxon) to identify lineages in the species tree under positive selection. We then used the datamonkey interface to specify those same branches as foreground branches in BUSTED models fitted to data for each alignment of egg-expressed genes (*Zp2*, *Zp3*, *Juno* in both taxa) that encode egg coat proteins that interact with sperm. We used those results to ask whether the BUSTED model with an additional class of positively selected *Zp2* (or *Zp3* or *Juno*) codons on those foreground branches was a significantly better fit to the *Zp2* (or *Zp3* or *Juno*) data, and thus an indication of coevolution of the pair of genes on the same subset of branches in the species tree.

Our preregistration included one BUSTED analysis that we did not carry out. We had planned a direct comparison of the sexual selection hypothesis for *C4BPA* evolution in Primates (expressed in innate immunity and expressed in sperm, and coevolving with zona pellucida genes) by aligning those sequences with *C4bpa* genes from Glires (expressed only in innate immunity), and testing the significance of a BUSTED model with all primate lineages in the foreground class (relative to a null model with both taxa in the same nonselected class of lineages). We did not carry out that analysis because much of the alignment (including the fifth and sixth sushi domains, as well as the 5′ and 3′ nonrepetitive regions) was of doubtful quality with many alignment gaps.

## Additional files

Additional file 1: **Appendix S1.** aBSREL and MEME output files, and summary of codon model analyses. (ZIP 2616 kb)

Additional file 2: **Appendix S2.** Input files for codon model analyses (alignments, Newick strings) in fasta format (ZIP 91 kb)

Additional file 3: **Appendix S3.** Summary of species names, accession numbers, and Newick strings used in codon model analyses (XLSX 51 kb)

## Data Availability

The sequence data analyzed in this study come from public databases (Ensembl; GenBank). All outputs from codon model analyses (Additional file 1: Appendix 1) of sequence alignments (Additional file 2: Appendix 2) based on data from publicly available sequence accessions (Additional file 3: Appendix 3) are available with the full-text of this article.

## Abbreviations

aBSREL: Adaptive branch-site random effects likelihood
BUSTED: Branch-site unrestricted statistical test for episodic diversification
C4bpa: Alpha subunit of C4b-binding protein
C4bpb: Beta subunit of C4b-binding protein
CCP: Complement control protein repeat
HARKing: Hypothesizing after results are known
LD: Linkage disequilibrium
Ma: Million years
MEME: Mixed-effects model of evolution
RCA: Regulator of complement activation
SNP: Single nucleotide polymorphism
Sp56: Sperm protein 56
ZP: Zona pellucida
Zp1: Zona pellucida protein 1
Zp2: Zona pellucida protein 2
Zp3: Zona pellucida protein 3
Zp3r: Zona pellucida 3 receptor
Zp4: Zona pellucida protein 4
