# Supplementary material for: Molecular evolution of mammalian genes with epistatic interactions in fertilization
Source: BMC Evol Biol. 2019 Jul 25;19:154. doi: 10.1186/s12862-019-1480-6 (PMC6659299; doi:10.1186/s12862-019-1480-6)
Supplement: Supplementary file 1 — Appendix S1. aBSREL and MEME output files, and summary of codon model analyses. (ZIP 2616 kb) [file 12862_2019_1480_MOESM1_ESM.zip › juno_Glires_aBSREL.htm]

Datamonkey Adaptive Evolution Server


- Methods and Tools 
  - aBSREL
  - BUSTED
  - FEL
  - FUBAR
  - GARD
  - HIV-TRACE
  - MEME
  - RELAX
  - SLAC
  - All Methods
- Job Queue
- Usage statistics
- Citations
- Help


- summary
- tree
- table
- model fits

×Close**Error!**

### adaptive Branch Site REL results summary

INPUT DATA |5b2fdde918ed6e609e246814|22 sequences |243 sites

Export

- Original file
- Analysis log
- Save JSON
- View JSON

aBSREL **found no evidence** of episodic diversifying selection in your phylogeny.

A total of **41** branches were formally tested for diversifying selection. Significance was assessed using the Likelihood Ratio Test at a threshold of p ≤ 0.05, after correcting for multiple testing. Significance and number of rate categories inferred at each branch are provided in the detailed results table.

---

See here for more information about the aBSREL method.  
Please cite PMID 25697341 if you use this result in a publication, presentation, or other scientific work.

#### Tree summary

| ω rate classes | # of branches | % of branches | % of tree length | # under selection |
| --- | --- | --- | --- | --- |
| 1 | 32 | 78% | 5.9% | 0 |
| 2 | 9 | 22% | 94% | 0 |

This table contains a summary of the inferred aBSREL model complexity. Each row provides information about the branches that were best described by the given number of ω rate categories.

#### Fitted tree

Options

- Models
- Full adaptive model
- Baseline MG94xREV

Linear Radial

Export 

- PNG
- SVG
- Newick File

- Hide Legend
- GrayScale

00.010.10.512510ωLength = 0.08450167085869024Length = 0.05704622788643574Length = 0.07362365135374313Length = 0.03756226090633105Length = 0.05865870147198158Length = 0.00615213403972356Length = 0.01957276181654423Length = 0.01080230161522563Length = 0.03795728753613969Length = 0.0179286039006681Length = 0.06841823781299176Length = 0.0552442149835144Length = 0.08330969711606301Length = 0.02035596725816074Length = 0.02316609237371997Length = 0.007949306459761845Length = 0.04729476039006685Length = 0.1604638898585398Length = 0.04942729155818033Length = 0.2631546003796442Length = 0.1457735991333964Length = 0.1198698459506866Length = 0.03640974345510812Length = 0.009983495677365376Length = 0.04789412678001636Length = 0.04878348698223667Length = 0.03862199277338393Length = 0.09825489118177809Length = 0.06914468673408102Length = 0.01249029988939796Length = 0.01042497952290521Length = 0.02651492442329026Length = 0.003732024872966986Length = 21.49592962157884Length = 0.008107302438045057Length = 0.01168933571604502Length = 0.01261374155825251Length = 0.09787212057668687Length = 0.1072647779100892Length = 0.04493285869402047Length = 0.1005479112437615PIKARABBITSQUIRRELMARMOTDEGULONG\_TAILED\_CHINCHILLAGUINEA\_PIGNAKED\_MOLE\_RATDAMARA\_MOLE\_RATBEAVERKANGAROO\_RATLESSER\_EGYPTIAN\_JERBOABLIND\_MOLE\_RATHOUSE\_MOUSERYUKYU\_MOUSESHREW\_MOUSERATMONGOLIAN\_GERBILCHINESE\_HAMSTERGOLDEN\_HAMSTERPRAIRIE\_VOLEDEER\_MOUSE2.04.06.08.0101214161820

#### Detailed results

| Name | B | LRT | Test p-value | Uncorrected p-value | ω distribution over sites |  |
| --- | --- | --- | --- | --- | --- | --- |
| Node10 | 0.0000 | 5.7158 | 0.8438 | 0.0206 | ω1 = 0.00 (93%) ω2 = 26.8 (7.1%) |  |
| CHINESE\_HAMSTER | 0.0000 | 5.4654 | 0.9357 | 0.0234 | ω1 = 0.00 (91%) ω2 = 7.61 (9.2%) |  |
| MONGOLIAN\_GERBIL | 0.0000 | 0.0000 | 1.0000 | 1.0000 | ω1 = 0.346 (100%) |  |
| LESSER\_EGYPTIAN\_JERBOA | 0.0000 | 3.0800 | 1.0000 | 0.0802 | ω1 = 0.149 (92%) ω2 = 6.35 (7.7%) |  |
| HOUSE\_MOUSE | 0.0000 | 0.0000 | 1.0000 | 1.0000 | ω1 = 0.778 (100%) |  |
| RYUKYU\_MOUSE | 0.0000 | 0.0381 | 1.0000 | 0.4634 | ω1 = 1.15 (100%) |  |
| SHREW\_MOUSE | 0.0000 | 0.0000 | 1.0000 | 1.0000 | ω1 = 0.697 (100%) |  |
| RAT | 0.0000 | 0.0000 | 1.0000 | 1.0000 | ω1 = 0.403 (100%) |  |
| DEER\_MOUSE | 0.0000 | 0.0000 | 1.0000 | 1.0000 | ω1 = 0.337 (100%) |  |
| PIKA | 0.0000 | 0.0000 | 1.0000 | 1.0000 | ω1 = 0.0925 (100%) |  |
| GOLDEN\_HAMSTER | 0.0000 | 0.0000 | 1.0000 | 1.0000 | ω1 = 0.292 (100%) |  |
| PRAIRIE\_VOLE | 0.0000 | 4.1900 | 1.0000 | 0.0451 | ω1 = 0.0576 (89%) ω2 = 4.89 (11%) |  |
| BLIND\_MOLE\_RAT | 0.0000 | 2.1504 | 1.0000 | 0.1309 | ω1 = 0.296 (97%) ω2 = 22.2 (2.6%) |  |
| NAKED\_MOLE\_RAT | 0.0000 | 0.0000 | 1.0000 | 1.0000 | ω1 = 0.255 (100%) |  |
| DAMARA\_MOLE\_RAT | 0.0000 | 0.0000 | 1.0000 | 1.0000 | ω1 = 0.155 (100%) |  |
| GUINEA\_PIG | 0.0000 | 0.0000 | 1.0000 | 1.0000 | ω1 = 0.216 (100%) |  |
| DEGU | 0.0000 | 0.0000 | 1.0000 | 1.0000 | ω1 = 0.329 (100%) |  |
| LONG\_TAILED\_CHINCHILLA | 0.0000 | 0.0000 | 1.0000 | 1.0000 | ω1 = 0.365 (100%) |  |
| SQUIRREL | 0.0000 | 0.0000 | 1.0000 | 1.0000 | ω1 = 0.294 (100%) |  |
| BEAVER | 0.0000 | 1.9501 | 1.0000 | 0.1457 | ω1 = 0.00 (77%) ω2 = 2.16 (23%) |  |
| RABBIT | 0.0000 | 0.0000 | 1.0000 | 1.0000 | ω1 = 0.146 (100%) |  |
| MARMOT | 0.0000 | 0.0000 | 1.0000 | 1.0000 | ω1 = 0.398 (100%) |  |
| KANGAROO\_RAT | 0.0000 | 0.0000 | 1.0000 | 1.0000 | ω1 = 0.284 (100%) |  |
| Node11 | 0.0000 | 0.0000 | 1.0000 | 1.0000 | ω1 = 0.203 (100%) |  |
| Node15 | 0.0000 | 0.0000 | 1.0000 | 1.0000 | ω1 = 0.994 (100%) |  |
| Node18 | 0.0000 | 0.2067 | 1.0000 | 0.4004 | ω1 = 10000000000 (100%) |  |
| Node19 | 0.0000 | 0.1194 | 1.0000 | 0.4286 | ω1 = 5.54 (100%) |  |
| Node22 | 0.0000 | 2.5858 | 1.0000 | 0.1039 | ω1 = 0.00 (95%) ω2 = 6.73 (4.6%) |  |
| Node24 | 0.0000 | 0.0000 | 1.0000 | 1.0000 | ω1 = 0.286 (100%) |  |
| Node26 | 0.0000 | 0.0000 | 1.0000 | 1.0000 | ω1 = 0.255 (100%) |  |
| Node27 | 0.0000 | 0.0000 | 1.0000 | 1.0000 | ω1 = 0.226 (100%) |  |
| Node28 | 0.0000 | 2.8892 | 1.0000 | 0.0886 | ω1 = 10000000000 (100%) |  |
| Node29 | 0.0000 | 0.0000 | 1.0000 | 1.0000 | ω1 = 0.523 (100%) |  |
| Node30 | 0.0000 | 0.0000 | 1.0000 | 1.0000 | ω1 = 0.172 (100%) |  |
| Node36 | 0.0000 | 0.0000 | 1.0000 | 1.0000 | ω1 = 0.342 (100%) |  |
| Node37 | 0.0000 | 1.3638 | 1.0000 | 0.2003 | ω1 = 10000000000 (100%) |  |
| Node38 | 0.0000 | 0.0000 | 1.0000 | 1.0000 | ω1 = 0.358 (100%) |  |
| Node4 | 0.0000 | 4.6468 | 1.0000 | 0.0356 | ω1 = 0.00 (83%) ω2 = 3.15 (17%) |  |
| Node5 | 0.0000 | 0.0000 | 1.0000 | 1.0000 | ω1 = 0.295 (100%) |  |
| Node8 | 0.0000 | 0.0000 | 1.0000 | 1.0000 | ω1 = 0.614 (100%) |  |
| Node9 | 0.0000 | 4.6520 | 1.0000 | 0.0355 | ω1 = 0.269 (98%) ω2 = 10000 (2.4%) |  |

×

#### aBSREL Site Proportion Chart

#### ω distribution

# **PIKA**

SVG PNG

Neutrality (ω=1)ω0.000010.00010.0010.010.1110100100010000Proportion of sites0%10%20%30%40%50%60%70%80%90%100%

Close

#### Model fits

| Model | AICC | log L | Parameters |
| --- | --- | --- | --- |
| Nucleotide GTR | 14124.89 | -7012.98 | 49 |
| Baseline MG94xREV | 13717.98 | -6761.21 | 96 |
| Full adaptive model | 13648.29 | -6707.64 | 114 |

This table reports a statistical summary of the models fit to the data. Here, **Baseline MG94xREV** refers to the MG94xREV baseline model that infers a single ω rate category per branch. **Full adaptive model** refers to the adaptive aBSREL model that infers an optimized number of ω rate categories per branch.

×

#### Error

This is my error message

Close

Datamonkey is funded jointly by MIDAS and NIH award R01 GM093939
