# Supplementary material for: Molecular evolution of mammalian genes with epistatic interactions in fertilization
Source: BMC Evol Biol. 2019 Jul 25;19:154. doi: 10.1186/s12862-019-1480-6 (PMC6659299; doi:10.1186/s12862-019-1480-6)
Supplement: Supplementary file 1 — Appendix S1. aBSREL and MEME output files, and summary of codon model analyses. (ZIP 2616 kb) [file 12862_2019_1480_MOESM1_ESM.zip › juno_Primates_aBSREL.htm]

Datamonkey Adaptive Evolution Server


- Methods and Tools 
  - aBSREL
  - BUSTED
  - FEL
  - FUBAR
  - GARD
  - HIV-TRACE
  - MEME
  - RELAX
  - SLAC
  - All Methods
- Job Queue
- Usage statistics
- Citations
- Help


- summary
- tree
- table
- model fits

×Close**Error!**

### adaptive Branch Site REL results summary

INPUT DATA |5b2fde2918ed6e609e246830|22 sequences |250 sites

Export

- Original file
- Analysis log
- Save JSON
- View JSON

aBSREL **found no evidence** of episodic diversifying selection in your phylogeny.

A total of **41** branches were formally tested for diversifying selection. Significance was assessed using the Likelihood Ratio Test at a threshold of p ≤ 0.05, after correcting for multiple testing. Significance and number of rate categories inferred at each branch are provided in the detailed results table.

---

See here for more information about the aBSREL method.  
Please cite PMID 25697341 if you use this result in a publication, presentation, or other scientific work.

#### Tree summary

| ω rate classes | # of branches | % of branches | % of tree length | # under selection |
| --- | --- | --- | --- | --- |
| 1 | 38 | 93% | 68% | 0 |
| 2 | 3 | 7.3% | 32% | 0 |

This table contains a summary of the inferred aBSREL model complexity. Each row provides information about the branches that were best described by the given number of ω rate categories.

#### Fitted tree

Options

- Models
- Full adaptive model
- Baseline MG94xREV

Linear Radial

Export 

- PNG
- SVG
- Newick File

- Hide Legend
- GrayScale

00.010.10.512510ωLength = 0.001347902637870855Length = 0Length = 0.006772392228393269Length = 0.00982265522174536Length = 0.006840366082294775Length = 0.002743383257530106Length = 0.001350144980362921Length = 0Length = 0.002734089003469531Length = 0Length = 0Length = 0.001349123151146731Length = 0.001348377085317891Length = 0.001367323456741294Length = 0.008427859633382433Length = 0.001385040435918424Length = 0.00280207641389618Length = 0.001347714430927758Length = 0Length = 0.002734710583143956Length = 0.002696155659868245Length = 0.001389976687355465Length = 0.01522565153308836Length = 0.007613104208635765Length = 0.01032406149612093Length = 0.001713629564532804Length = 0.007543197819025509Length = 0.006311969499481648Length = 0.01567936561708335Length = 0.01625684439934789Length = 0.01854815665609132Length = 0.02115043938067392Length = 0.003657045119408298Length = 0.002308262806870869Length = 0.0201081072241743Length = 0.01843460489048193Length = 0.01074377147798559Length = 0.05920327904941453Length = 0.09345741659378691Length = 0.06046271883212092Length = 0.1043226117839102BUSHBABYMOUSE\_LEMURCOQUERELS\_SIFAKABOLIVIAN\_SQUIRREL\_MONKEYCAPUCHINMAS\_NIGHT\_MONKEYMARMOSETHUMANBONOBOGORILLAORANGUTANGIBBONDRILLOLIVE\_BABOONMACAQUECRAB\_EATING\_MACAQUEPIG\_TAILED\_MACAQUEVERVETRED\_COLOBUSANGOLA\_COLOBUSBLACK\_SNUB\_NOSED\_MONKEYGOLDEN\_SNUB\_NOSED\_MONKEY0.0200.0400.0600.0800.100.12

#### Detailed results

| Name | B | LRT | Test p-value | Uncorrected p-value | ω distribution over sites |  |
| --- | --- | --- | --- | --- | --- | --- |
| Node6 | 0.0000 | 6.9637 | 0.4464 | 0.0109 | ω1 = 0.00 (88%) ω2 = 6.00 (12%) |  |
| VERVET | 0.0000 | 0.0000 | 1.0000 | 1.0000 | ω1 = 0.652 (100%) |  |
| BLACK\_SNUB\_NOSED\_MONKEY | 0.0000 | 0.0000 | 1.0000 | 1.0000 | ω1 = 1.00 (100%) |  |
| BOLIVIAN\_SQUIRREL\_MONKEY | 0.0000 | 0.0000 | 1.0000 | 1.0000 | ω1 = 0.317 (100%) |  |
| BONOBO | 0.0000 | 0.0000 | 1.0000 | 1.0000 | ω1 = 1.00 (100%) |  |
| BUSHBABY | 0.0000 | 0.0000 | 1.0000 | 1.0000 | ω1 = 0.296 (100%) |  |
| CAPUCHIN | 0.0000 | 0.0000 | 1.0000 | 1.0000 | ω1 = 0.302 (100%) |  |
| COQUERELS\_SIFAKA | 0.0000 | 0.0000 | 1.0000 | 1.0000 | ω1 = 0.374 (100%) |  |
| CRAB\_EATING\_MACAQUE | 0.0000 | 0.5691 | 1.0000 | 0.3158 | ω1 = 10000000000 (100%) |  |
| DRILL | 0.0000 | 0.5721 | 1.0000 | 0.3153 | ω1 = 10000000000 (100%) |  |
| GIBBON | 0.0000 | 0.0000 | 1.0000 | 1.0000 | ω1 = 0.216 (100%) |  |
| GOLDEN\_SNUB\_NOSED\_MONKEY | 0.0000 | 0.5758 | 1.0000 | 0.3145 | ω1 = 10000000000 (100%) |  |
| GORILLA | 0.0000 | 1.0228 | 1.0000 | 0.2423 | ω1 = 10000000000 (100%) |  |
| MAS\_NIGHT\_MONKEY | 0.0000 | 0.0000 | 1.0000 | 1.0000 | ω1 = 0.830 (100%) |  |
| MACAQUE | 0.0000 | 0.0000 | 1.0000 | 1.0000 | ω1 = 1.00 (100%) |  |
| MARMOSET | 0.0000 | 0.0000 | 1.0000 | 1.0000 | ω1 = 0.692 (100%) |  |
| MOUSE\_LEMUR | 0.0000 | 0.0000 | 1.0000 | 1.0000 | ω1 = 0.235 (100%) |  |
| OLIVE\_BABOON | 0.0000 | 0.0000 | 1.0000 | 1.0000 | ω1 = 1.00 (100%) |  |
| ORANGUTAN | 0.0000 | 0.0000 | 1.0000 | 1.0000 | ω1 = 0.192 (100%) |  |
| PIG\_TAILED\_MACAQUE | 0.0000 | 0.0000 | 1.0000 | 1.0000 | ω1 = 0.325 (100%) |  |
| ANGOLA\_COLOBUS | 0.0000 | 0.0777 | 1.0000 | 0.4447 | ω1 = 1.37 (100%) |  |
| RED\_COLOBUS | 0.0000 | 0.0000 | 1.0000 | 1.0000 | ω1 = 0.0530 (100%) |  |
| Node11 | 0.0000 | 0.0000 | 1.0000 | 1.0000 | ω1 = 0.00 (100%) |  |
| Node14 | 0.0000 | 3.0372 | 1.0000 | 0.0820 | ω1 = 0.00 (97%) ω2 = 12.2 (2.9%) |  |
| Node15 | 0.0000 | 0.0000 | 1.0000 | 1.0000 | ω1 = 0.241 (100%) |  |
| Node16 | 0.0000 | 0.0000 | 1.0000 | 1.0000 | ω1 = 0.00 (100%) |  |
| Node17 | 0.0000 | 0.0000 | 1.0000 | 1.0000 | ω1 = 0.184 (100%) |  |
| Node18 | 0.0000 | 0.0000 | 1.0000 | 1.0000 | ω1 = 0.00 (100%) |  |
| Node24 | 0.0000 | 0.0000 | 1.0000 | 1.0000 | ω1 = 0.501 (100%) |  |
| Node25 | 0.0000 | 0.2497 | 1.0000 | 0.3883 | ω1 = 10000000000 (100%) |  |
| Node26 | 0.0000 | 0.0000 | 1.0000 | 1.0000 | ω1 = 0.00 (100%) |  |
| Node27 | 0.0000 | 0.0000 | 1.0000 | 1.0000 | ω1 = 0.00 (100%) |  |
| Node3 | 0.0000 | 0.9850 | 1.0000 | 0.2475 | ω1 = 0.00 (92%) ω2 = 2.69 (8.5%) |  |
| Node30 | 0.0000 | 0.5639 | 1.0000 | 0.3168 | ω1 = 10000000000 (100%) |  |
| Node31 | 0.0000 | 0.0000 | 1.0000 | 1.0000 | ω1 = 1.00 (100%) |  |
| Node36 | 0.0000 | 0.0000 | 1.0000 | 1.0000 | ω1 = 0.00 (100%) |  |
| Node37 | 0.0000 | 0.0000 | 1.0000 | 1.0000 | ω1 = 0.325 (100%) |  |
| Node40 | 0.0000 | 0.0000 | 1.0000 | 1.0000 | ω1 = 0.488 (100%) |  |
| HUMAN | 0.0000 | 0.0000 | 1.0000 | 1.0000 | ω1 = 0.323 (100%) |  |
| Node7 | 0.0000 | 0.0000 | 1.0000 | 1.0000 | ω1 = 0.673 (100%) |  |
| Node8 | 0.0000 | 0.0000 | 1.0000 | 1.0000 | ω1 = 0.00 (100%) |  |

×

#### aBSREL Site Proportion Chart

#### ω distribution

# **HUMAN**

SVG PNG

Neutrality (ω=1)ω0.000010.00010.0010.010.1110100100010000Proportion of sites0%10%20%30%40%50%60%70%80%90%100%

Close

#### Model fits

| Model | AICC | log L | Parameters |
| --- | --- | --- | --- |
| Nucleotide GTR | 5685.08 | -2793.09 | 49 |
| Baseline MG94xREV | 5537.89 | -2671.22 | 96 |
| Full adaptive model | 5520.90 | -2656.50 | 102 |

This table reports a statistical summary of the models fit to the data. Here, **Baseline MG94xREV** refers to the MG94xREV baseline model that infers a single ω rate category per branch. **Full adaptive model** refers to the adaptive aBSREL model that infers an optimized number of ω rate categories per branch.

×

#### Error

This is my error message

Close

Datamonkey is funded jointly by MIDAS and NIH award R01 GM093939
