# Supplementary material for: Molecular evolution of mammalian genes with epistatic interactions in fertilization
Source: BMC Evol Biol. 2019 Jul 25;19:154. doi: 10.1186/s12862-019-1480-6 (PMC6659299; doi:10.1186/s12862-019-1480-6)
Supplement: Supplementary file 1 — Appendix S1. aBSREL and MEME output files, and summary of codon model analyses. (ZIP 2616 kb) [file 12862_2019_1480_MOESM1_ESM.zip › zp3_Primates_aBSREL.htm]

Datamonkey Adaptive Evolution Server


- Methods and Tools 
  - aBSREL
  - BUSTED
  - FEL
  - FUBAR
  - GARD
  - HIV-TRACE
  - MEME
  - RELAX
  - SLAC
  - All Methods
- Job Queue
- Usage statistics
- Citations
- Help


- summary
- tree
- table
- model fits

×Close**Error!**

### adaptive Branch Site REL results summary

INPUT DATA |5b2fdfa218ed6e609e24690b|25 sequences |424 sites

Export

- Original file
- Analysis log
- Save JSON
- View JSON

aBSREL **found no evidence** of episodic diversifying selection in your phylogeny.

A total of **47** branches were formally tested for diversifying selection. Significance was assessed using the Likelihood Ratio Test at a threshold of p ≤ 0.05, after correcting for multiple testing. Significance and number of rate categories inferred at each branch are provided in the detailed results table.

---

See here for more information about the aBSREL method.  
Please cite PMID 25697341 if you use this result in a publication, presentation, or other scientific work.

#### Tree summary

| ω rate classes | # of branches | % of branches | % of tree length | # under selection |
| --- | --- | --- | --- | --- |
| 1 | 41 | 87% | 45% | 0 |
| 2 | 6 | 13% | 55% | 0 |

This table contains a summary of the inferred aBSREL model complexity. Each row provides information about the branches that were best described by the given number of ω rate categories.

#### Fitted tree

Options

- Models
- Full adaptive model
- Baseline MG94xREV

Linear Radial

Export 

- PNG
- SVG
- Newick File

- Hide Legend
- GrayScale

00.010.10.512510ωLength = 0.0008379666659176671Length = 0.0009114987913170701Length = 0.00447157247677134Length = 0.006439731757929785Length = 0.009953400759206123Length = 0.001842649643181017Length = 0.00158436598041259Length = 0.0007899232045495749Length = 0.001581026999388565Length = 0Length = 0.001962838627357134Length = 0.003974936499326243Length = 0.006377300498460543Length = 0Length = 0.001580701125432135Length = 0Length = 0.0076521574867061Length = 0.007222955690416981Length = 0Length = 0.003582378038446677Length = 0.003566043409562277Length = 0.0007913063137066382Length = 0.00157591263037445Length = 0.01304564439722706Length = 0.002336386919101195Length = 0.0009195236550403547Length = 0.01613935005547614Length = 0.008042720663481608Length = 0.07565138712533079Length = 0Length = 0.0203575700558792Length = 0.005451016904987077Length = 0.02523420929544554Length = 0.01304804136697321Length = 0.009284970829689673Length = 0.01761524778594794Length = 0Length = 0.004129294148204878Length = 0.01652683686409869Length = 0.03674877611948681Length = 0.2317366037061097Length = 0.09572354628200681Length = 0.03634299545927282Length = 0.04646657170342317Length = 0.04767480962230652Length = 0.05275468314420742Length = 0.1019202393478616BUSHBABYMOUSE\_LEMURCOQUERELS\_SIFAKATARSIERBOLIVIAN\_SQUIRREL\_MONKEYCAPUCHINMAS\_NIGHT\_MONKEYMARMOSETHUMANCHIMPANZEEBONOBOGORILLAORANGUTANGIBBONSOOTY\_MANGABEYDRILLOLIVE\_BABOONMACAQUECRAB\_EATING\_MACAQUEPIG\_TAILED\_MACAQUEVERVETRED\_COLOBUSANGOLA\_COLOBUSBLACK\_SNUB\_NOSED\_MONKEYGOLDEN\_SNUB\_NOSED\_MONKEY0.0500.100.150.200.250.300.35

#### Detailed results

| Name | B | LRT | Test p-value | Uncorrected p-value | ω distribution over sites |  |
| --- | --- | --- | --- | --- | --- | --- |
| HUMAN | 0.0000 | 8.4441 | 0.2413 | 0.0051 | ω1 = 1.00 (100%) ω2 = 1060 (0.32%) |  |
| Node9 | 0.0000 | 0.0000 | 1.0000 | 1.0000 | ω1 = 0.356 (100%) |  |
| BLACK\_SNUB\_NOSED\_MONKEY | 0.0000 | 0.6309 | 1.0000 | 0.3043 | ω1 = 10000000000 (100%) |  |
| BOLIVIAN\_SQUIRREL\_MONKEY | 0.0000 | 0.0000 | 1.0000 | 1.0000 | ω1 = 0.141 (100%) |  |
| BONOBO | 0.0000 | 0.0000 | 1.0000 | 1.0000 | ω1 = 0.385 (100%) |  |
| BUSHBABY | 0.0000 | 0.0000 | 1.0000 | 1.0000 | ω1 = 0.202 (100%) |  |
| CAPUCHIN | 0.0000 | 0.0000 | 1.0000 | 1.0000 | ω1 = 0.402 (100%) |  |
| CHIMPANZEE | 0.0000 | 0.6503 | 1.0000 | 0.3007 | ω1 = 10000000000 (100%) |  |
| COQUERELS\_SIFAKA | 0.0000 | 0.0000 | 1.0000 | 1.0000 | ω1 = 0.278 (100%) |  |
| CRAB\_EATING\_MACAQUE | 0.0000 | 0.0000 | 1.0000 | 1.0000 | ω1 = 0.00 (100%) |  |
| DRILL | 0.0000 | 0.0000 | 1.0000 | 1.0000 | ω1 = 0.00 (100%) |  |
| GIBBON | 0.0000 | 5.4762 | 1.0000 | 0.0233 | ω1 = 0.272 (100%) ω2 = 1760 (0.35%) |  |
| GOLDEN\_SNUB\_NOSED\_MONKEY | 0.0000 | 0.6437 | 1.0000 | 0.3019 | ω1 = 10000000000 (100%) |  |
| GORILLA | 0.0000 | 0.0000 | 1.0000 | 1.0000 | ω1 = 0.375 (100%) |  |
| MAS\_NIGHT\_MONKEY | 0.0000 | 0.0000 | 1.0000 | 1.0000 | ω1 = 0.222 (100%) |  |
| MACAQUE | 0.0000 | 0.6501 | 1.0000 | 0.3008 | ω1 = 10000000000 (100%) |  |
| MARMOSET | 0.0000 | 0.0000 | 1.0000 | 1.0000 | ω1 = 0.234 (100%) |  |
| MOUSE\_LEMUR | 0.0000 | 0.0000 | 1.0000 | 1.0000 | ω1 = 0.394 (100%) |  |
| OLIVE\_BABOON | 0.0000 | 0.0000 | 1.0000 | 1.0000 | ω1 = 0.127 (100%) |  |
| ORANGUTAN | 0.0000 | 0.0000 | 1.0000 | 1.0000 | ω1 = 0.0929 (100%) |  |
| PIG\_TAILED\_MACAQUE | 0.0000 | 0.0000 | 1.0000 | 1.0000 | ω1 = 0.00 (100%) |  |
| SOOTY\_MANGABEY | 0.0000 | 0.0000 | 1.0000 | 1.0000 | ω1 = 0.570 (100%) |  |
| TARSIER | 0.0000 | 0.3356 | 1.0000 | 0.3661 | ω1 = 0.00 (72%) ω2 = 1.27 (28%) |  |
| ANGOLA\_COLOBUS | 0.0000 | 0.0000 | 1.0000 | 1.0000 | ω1 = 0.374 (100%) |  |
| RED\_COLOBUS | 0.0000 | 0.0000 | 1.0000 | 1.0000 | ω1 = 0.157 (100%) |  |
| Node10 | 0.0000 | 0.0000 | 1.0000 | 1.0000 | ω1 = 0.00 (100%) |  |
| Node13 | 0.0000 | 0.0000 | 1.0000 | 1.0000 | ω1 = 1.00 (100%) |  |
| Node16 | 0.0000 | 0.0000 | 1.0000 | 1.0000 | ω1 = 0.133 (100%) |  |
| Node17 | 0.0000 | 0.0000 | 1.0000 | 1.0000 | ω1 = 0.428 (100%) |  |
| Node18 | 0.0000 | 0.0000 | 1.0000 | 1.0000 | ω1 = 1.00 (100%) |  |
| Node19 | 0.0000 | 0.0000 | 1.0000 | 1.0000 | ω1 = 0.294 (100%) |  |
| Node20 | 0.0000 | 0.0000 | 1.0000 | 1.0000 | ω1 = 0.00 (100%) |  |
| Node22 | 0.0000 | 0.0000 | 1.0000 | 1.0000 | ω1 = 0.00 (100%) |  |
| Node28 | 0.0000 | 0.0000 | 1.0000 | 1.0000 | ω1 = 0.360 (100%) |  |
| Node29 | 0.0000 | 0.0000 | 1.0000 | 1.0000 | ω1 = 0.147 (100%) |  |
| Node3 | 0.0000 | 3.0460 | 1.0000 | 0.0816 | ω1 = 0.0123 (88%) ω2 = 4.28 (12%) |  |
| Node30 | 0.0000 | 0.0000 | 1.0000 | 1.0000 | ω1 = 0.272 (100%) |  |
| Node31 | 0.0000 | 0.0000 | 1.0000 | 1.0000 | ω1 = 1.00 (100%) |  |
| Node32 | 0.0000 | 0.0000 | 1.0000 | 1.0000 | ω1 = 1.00 (100%) |  |
| Node36 | 0.0000 | 0.0000 | 1.0000 | 1.0000 | ω1 = 0.384 (100%) |  |
| Node37 | 0.0000 | 0.0000 | 1.0000 | 1.0000 | ω1 = 1.00 (100%) |  |
| Node42 | 0.0000 | 0.0000 | 1.0000 | 1.0000 | ω1 = 1.00 (100%) |  |
| Node43 | 0.0000 | 0.8027 | 1.0000 | 0.2749 | ω1 = 10000000000 (100%) |  |
| Node46 | 0.0000 | 0.0000 | 1.0000 | 1.0000 | ω1 = 0.369 (100%) |  |
| Node6 | 0.0000 | 5.3436 | 1.0000 | 0.0249 | ω1 = 0.481 (98%) ω2 = 60.4 (1.8%) |  |
| Node8 | 0.0000 | 1.8524 | 1.0000 | 0.1535 | ω1 = 0.274 (99%) ω2 = 231 (0.74%) |  |
| VERVET | 0.0000 | 0.0000 | 1.0000 | 1.0000 | ω1 = 0.300 (100%) |  |

×

#### aBSREL Site Proportion Chart

#### ω distribution

# **HUMAN**

SVG PNG

Neutrality (ω=1)ω0.000010.00010.0010.010.1110100100010000Proportion of sites0%10%20%30%40%50%60%70%80%90%100%

Close

#### Model fits

| Model | AICC | log L | Parameters |
| --- | --- | --- | --- |
| Nucleotide GTR | 11431.94 | -5660.68 | 55 |
| Baseline MG94xREV | 11157.08 | -5469.42 | 108 |
| Full adaptive model | 11127.38 | -5442.30 | 120 |

This table reports a statistical summary of the models fit to the data. Here, **Baseline MG94xREV** refers to the MG94xREV baseline model that infers a single ω rate category per branch. **Full adaptive model** refers to the adaptive aBSREL model that infers an optimized number of ω rate categories per branch.

×

#### Error

This is my error message

Close

Datamonkey is funded jointly by MIDAS and NIH award R01 GM093939
